# The Narrow Abdomen Ion Channel Complex Is Highly Stable and Persists from Development into Adult Stages to Promote Behavioral Rhythmicity

**DOI:** 10.3389/fncel.2017.00159

**Published:** 2017-06-06

**Authors:** Devon L. Moose, Stephanie J. Haase, Benjamin T. Aldrich, Bridget C. Lear

**Affiliations:** ^1^Department of Biology, University of Iowa, Iowa CityIA, United States; ^2^Interdisciplinary Graduate Program in Genetics, University of Iowa, Iowa CityIA, United States

**Keywords:** circadian rhythms, ion channel, NALCN, *Drosophila melanogaster*, Nlf-1, narrow abdomen

## Abstract

The sodium leak channel NARROW ABDOMEN (NA)/ NALCN is an important component of circadian pacemaker neuronal output. In *Drosophila*, rhythmic expression of the NA channel regulator *Nlf-1* in a subset of adult pacemaker neurons has been proposed to contribute to circadian regulation of channel localization or activity. Here we have restricted expression of *Drosophila* NA channel subunits or the *Nlf-1* regulator to either development or adulthood using the temperature-inducible *tubulin*-GAL80^ts^ system. Surprisingly, we find that developmental expression of endogenous channel subunits and *Nlf-1* is sufficient to promote robust rhythmic behavior in adults. Moreover, we find that channel complex proteins produced during development persist in the *Drosophila* head with little decay for at least 5–7 days in adults. In contrast, restricting either endogenous or transgenic gene expression to adult stages produces only limited amounts of the functional channel complex. These data indicate that much of the NA channel complex that functions in adult circadian neurons is normally produced during development, and that the channel complex is very stable in most neurons in the *Drosophila* brain. Based on these findings, we propose that circadian regulation of NA channel function in adult pacemaker neurons is mediated primarily by post-translational mechanisms that are independent of *Nlf-1.*

## Introduction

Circadian clocks promote daily rhythms in animal behavior and physiology. The molecular circadian clock mechanism consists of transcriptional and translational feedback loops that produce ∼24 h rhythms in gene expression (Allada and Chung, 2010; Partch et al., 2014). In both insects and mammals, clock-containing neurons in the brain drive daily rhythms in locomotor behavior. These circadian clock neurons exhibit rhythms in resting membrane potential and firing frequency to promote rhythmic outputs. Yet while the mechanisms that produce ∼24 h molecular clock oscillations have been well characterized, the mechanisms that regulate daily neuronal activity rhythms are not as well understood (Colwell, 2011). In mammals, the suprachiasmatic nucleus (SCN) in the hypothalamus acts as a master circadian pacemaker to synchronize clocks within other tissues and promote daily behavioral rhythms (Mohawk et al., 2012). In *Drosophila*, small clusters of circadian pacemaker neurons in the adult brain drive a crepuscular behavioral pattern, with peaks of locomotor activity near dawn and dusk. Specific subsets of *Drosophila* circadian neurons, including the small ventral lateral neurons (s-LNv) and a group of posterior dorsal neurons (DN1p), are primarily implicated in the regulation of locomotor activity during morning hours. A separate subset of circadian neurons in the *Drosophila* brain, the lateral dorsal neurons (LNd), contributes prominently to the evening activity peak (Allada and Chung, 2010; Zhang L. et al., 2010; Zhang Y. et al., 2010). Circadian neurons also communicate with each other to synchronize clock oscillations and promote robust rhythmic output (Herzog, 2007). In *Drosophila*, signaling between or within circadian neuron subgroups via the neuropeptide PIGMENT DISPERSING FACTOR (PDF) and its receptor (PDFR) impacts the expression and/or function of clock components through multiple mechanisms, including effects on neuronal activity and activity-independent signaling (Guo et al., 2014; Seluzicki et al., 2014; Mezan et al., 2016; Sabado et al., 2017). In the mammalian SCN, signaling between neurons expressing VASOACTIVE INTESTINAL PEPTIDE (VIP) and those expressing its receptor (VPAC2) also promotes clock synchrony, likely through effects on clock gene transcription (Hastings et al., 2014). Thus, circadian neuronal output has a bidirectional relationship with the molecular circadian clock. However, the extent to which neuronal output mechanisms and intercellular signaling processes vary among circadian neurons is not fully understood.

Within circadian neurons, one mechanism that promotes rhythmic output is clock regulation of ion channel expression/function. In both *Drosophila* and mouse, the calcium-activated potassium channel BK exhibits daily expression rhythms, and mammalian BK has been shown to contribute to daily rhythms in circadian neuron excitability (Ceriani et al., 2002; Meredith et al., 2006; Whitt et al., 2016). Several additional channels exhibit rhythmic expression in *Drosophila* and/or mammalian brain pacemakers, including L-type and T-type calcium channels and inward rectifier potassium channels (Colwell, 2011; Ruben et al., 2012). The sodium leak channel NARROW ABDOMEN (NA)/NALCN is also a key contributor to circadian neuronal output in both insects and mammals. This channel functions broadly in the central nervous system to depolarize resting membrane potential and promote excitability (Ren, 2011; Flourakis et al., 2015). Disruptions in human NALCN gene function are associated with severe neurodevelopmental conditions, including developmental delay, hypotonia, and intellectual disability (Cochet-Bissuel et al., 2014; Aoyagi et al., 2015; Chong et al., 2015; Gal et al., 2016). In *Drosophila*, NA channel function is required broadly in the circadian pacemaker network to promote robust daily behavioral rhythms (Lear et al., 2005). Electrophysiological data from *Drosophila* clock neuron subgroups (DN1p, large LNv) as well as the mammalian SCN indicate that loss of NA/NALCN function hyperpolarizes and silences circadian pacemaker neurons (Flourakis et al., 2015). NA/NALCN sodium leak current is also circadianly regulated in at least a subset of *Drosophila* and mammalian clock neurons, contributing to the observed daily rhythm in membrane potential within these cells (Flourakis et al., 2015).

Several mechanisms of NA/NALCN channel regulation have been described (Ren, 2011). The auxiliary NA/NALCN channel subunits UNC79 and UNC80 are required to maintain proper expression, localization, and/or function of the channel complex across multiple animal species (Cochet-Bissuel et al., 2014). In *Drosophila*, NA, UNC79, and UNC80 exhibit an interdependent, post-transcriptional regulatory relationship in which loss of one subunit causes decreased expression of all three proteins (Lear et al., 2013). NCA- localization factor 1 (*Nlf-1;* aka *mid1*) (Ghezzi et al., 2014) has also been identified as an important channel regulator. In *C. elegans*, NLF-1 functions in the endoplasmic reticulum to promote proper localization of the channel complex to the plasma membrane (Xie et al., 2013). In *Drosophila*, NLF-1 is required to maintain appropriate expression and function of the NA channel complex, although the subcellular location of *Drosophila* NLF-1 function is not known (Flourakis et al., 2015). *Nlf-1* transcript is rhythmically expressed in the DN1p subset of clock neurons in the adult *Drosophila* brain, suggesting that this mediates daily rhythms in NA channel function (Flourakis et al., 2015). If *Drosophila* NLF-1 is localized to the ER, it would be expected to play a transient role during the production of the NA channel complex. Thus for *Nlf-1* rhythms to contribute to rhythmic channel activity, this would likely require the half-life of the functional NA channel complex to be relatively short. Notably, previous attempts to identify daily rhythms in channel complex expression have been unsuccessful (Nash et al., 2002; Lear et al., 2005).

Here we examine the temporal requirements for expression of *Drosophila* NA channel subunits as well as NLF-1 using inducible transgenic RNA interference and rescue approaches. We find that developmental expression of endogenous channel components and the NLF-1 regulator can promote substantial rhythmicity in adults. We also observe that the channel complex is highly stable in the *Drosophila* head, persisting for five or more days after production with little decay. Our data indicate that much of the endogenous NA channel complex that normally functions in the adult *Drosophila* brain, including the circadian pacemaker, is produced during development. Interestingly, we find that adult-driven transgenic *na* expression can promote robust behavioral rhythmicity, indicating that developmental loss of *na* does not permanently compromise neuronal function. Moreover, our data indicate that adult production of the endogenous channel complex may contribute in some contexts, including within the pacemaker neurons that promote morning behavior.

## Materials and Methods

### *Drosophila* Strains and Cross Schemes

Short-hairpin RNA interference (RNAi) strains targeting *unc79* (HMC03213) and *Nlf-1* (HMS03014) were generated by the *Drosophila* RNAi Screening Center (Ni et al., 2011). We generated *na* short hairpin RNAi (termed *na139* RNAi) by annealing forward (5′-CTA GCA GTG GTG CAG CTA GAA ACA AAT AGT TAT ATT CAA GCA TAT TTG TTT CTA GCA GCT GCA CCG CG-3′) and reverse (5′-AAT TCG CGG TGC AGC TGC TAG AAA CAA ATA TGC TTG AAT ATA ACT ATT TGT TTC TAG CAG CTG CAC CAC TG-3′) oligonucleotides corresponding to the 5′ UTR of *na* transcript (Integrated DNA Technologies). The annealed product was cloned into the p-VALIUM20 vector (Ni et al., 2011) and introduced into the *attP2* locus using PhiC-31 integration (Bestgene Inc.). Other *Drosophila* strains have been previously described: *tubulin*GAL80^ts^ (McGuire et al., 2003), *daughterless*-GAL4 (Aldrich et al., 2010), *Clock8.0*GAL4 (Glossop et al., 2003), *elav*GAL4 (Lin and Goodman, 1994), UAS-*na* U4 (Lear et al., 2005), UAS-*Nlf-1-V5* (Flourakis et al., 2015), and *na^har^* (Nash et al., 2002).

For RNAi experiments, *daughterless*-GAL4; *tubulin*-GAL80^ts^ females were crossed to males containing either UAS-*RNAi* or control insertions (*attP2* or *attP40*) (Groth et al., 2004), and crosses were maintained at either 29°C or 19°C. For developmental knockdown experiments, progeny from 29°C crosses were transferred to 19°C within 2 days of eclosion, 2–7 days prior to the start of the 19°C behavioral assay in order to minimize concerns over RNAi persistence (Bosch et al., 2016). Adult-specific knockdown crosses were raised at 19°C and 0–8 day old progeny were shifted to 29°C on the 1st day of the behavioral assay. For ‘non-shifted’ conditions (19°C to 19°C or 29°C to 29°C), 0–8 day old progeny were loaded into behavioral assays. In NA rescue experiments, *na^har^* GAL4 females were crossed to *tub*GAL80^ts^ and/or UAS-*na* males. In some experiments, males also contained UAS-*Nlf-1* RNAi or UAS-*Nlf-1-V5* transgenes (see Results). Crosses were again raised at either 19°C or 29°C. For development-specific rescue experiments, 0–2 day old 29°C progeny were shifted to 19°C 0–6 days prior to the behavioral assay. For adult-specific behavioral rescue experiments, 0–8 day old 19°C progeny were shifted to 29°C on the day of the behavior assay. Progeny loaded into non-shifted rescue experiments (19°C to 19°C and 29°C to 29°C) were 0–8 days old.

### Behavioral Assays and Analyses

For 19°C behavioral assays, locomotor activity levels of adult male *Drosophila* were measured for 5 days of 12 h light: 12 h dark conditions followed by 7 days constant darkness (DD) using the *Drosophila* Activity Monitor system (Trikinetics). For 29°C assays, activity levels were measured for 5–6 days of 14 h light: 10 h dark conditions followed by 7 days DD. For DD rhythmicity analyses, chi-squared periodogram analyses were performed on individual flies over 7 days using ClockLab analysis software (Actimetrics). Flies were considered rhythmic if the chi-squared power was > = 10 above significance, using a 0.01 confidence interval (Lear et al., 2005). To produce light:dark (LD) activity profiles, activity levels of individual flies were normalized and averaged within genotypes over the last 4 days of LD conditions. In one experiment, the four LD days analyzed were non-consecutive due to a ∼12 h disruption in data collection (see Figure legends). LD-DD daily activity profiles include normalized data from the last day of LD conditions followed by the first 3 days of DD conditions. LD and daily activity profiles were generated using the Excel-based program Counting Macro (Pfeiffenberger et al., 2010). Morning activity index (MI) and evening activity index (EI) were calculated from LD data by determining the largest 2–4 h increase in normalized average activity of each genotype over the last 5 h of dark phase (MI) or the last 7 h of light phase (EI) (Lear et al., 2013). For DD MI and EI calculations, normalized activity levels were averaged over three consecutive 30-min time points. For MI, maximum average activity of each genotype was then determined for three consecutive 30-min time points over the 8 h (DD Day 1) or 9 h (DD Day 2) surrounding Circadian Time (CT) 0. For EI, maximum average activity was determined for five consecutive 30-min bins over the 10 h (DD Day 1) or 12 h (DD Day 2) surrounding CT 12. In 29°C assays, the minimum average activity over a 3-h period was determined both before and after the observed maximal morning or evening activity peak. MI and EI were then obtained by subtracting the average of these minimum values from the corresponding maximum activity value (Lear et al., 2009). In 19°C assays, control strains exhibit a less consistent midday decrease in activity in DD conditions than what is observed at 29°C (Majercak et al., 1999). Therefore, we determined the minimum morning activity averaged over a 4-h period preceding morning peak, and the minimum evening activity averaged over a 4-h period following the evening peak.

### Antibodies and Western Blot

Western blots were performed using protein extracts from adult *Drosophila* heads obtained during mixed light phase conditions (Zeitgeber Time 0–10 h), using methods described previously (Lear et al., 2013). For adult or developmentally restricted RNAi experiments, 0–2 day old adults were shifted from the permissive (29°C) or restrictive (19°C) temperature to the opposing condition for at least 7 days prior to extraction. Equal amounts of protein were loaded onto each gel (5–6 μg for NA and 10 μg for UNC79 gels), as determined by Bradford assay (Bio-Rad). A minimum of two biological replicates with varying lane order were performed. Protein expression levels were quantified using NIH ImageJ, with the intensity of the bands standardized to the average intensity of samples (Lear et al., 2013). To estimate protein half-life, we used data from four independent experiments in which *elav*GAL4 *na^har^*; *tub*GAL80^ts^/+; UAS*-na*/+ flies were exposed to the following conditions in parallel: (1) 29°C development – 29°C adult, (2) 29°C development shifted to 19°C for 5–7 days adulthood, and (3) 19°C development- 19°C adult. For these calculations, the band intensity within each experiment was standardized to the average intensity of samples 1–3 above. Protein half-life was calculated using the formula *N*_t_ = *N*_0_ (1/2)^t/t1/2,^ where N_0_ is the average standardized protein level in the 29°C – 29°C condition, N_t_ is the average protein level at time t at 19°C (*t* = 6, averaged from 5 to 7 days), and t_1/2_ is the half-life estimate.

### Statistical Analyses

For DD rhythmicity data, the proportion of rhythmic flies was determined as described above, and genotypes were compared using Fisher’s exact test. LD and DD activity index values (MI/EI) were compared between two genotypes by unpaired Student’s *t*-test, or among three or more genotypes using ANOVA followed by Dunnett’s multiple comparison test (Graphpad Prism). For protein expression comparisons, significance was determined using unpaired Student’s *t*-test (Microsoft Excel).

## Results

### Developmental Expression of Endogenous NA Channel Subunits Is Sufficient for Rhythmic Behavior in Adults

**FIGURE 1:**
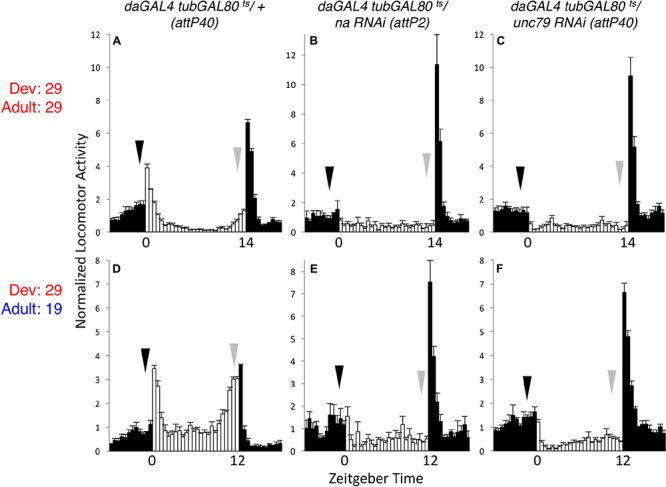
Developmental knockdown of *na* or *unc79* disrupts evening behavior in adults. Normalized activity patterns of adult male *Drosophila* averaged over 4 days of light:dark (LD) entrainment conditions. Zeitgeber time indicated below each panel. White bars represent activity levels during light phase, while black bars indicate activity during dark phase. Arrows indicate normal timing of morning (black) and evening (gray) activity increases, and error bars represent standard error of the mean. **(A–C)** *daGAL4 tubGAL80^ts^* genotypes raised at 29°C and maintained at 29°C throughout the behavioral assay. For 29°C assays, flies were subject to 14 h light: 10 h dark conditions. **(A)** *attP40* RNAi insertion control (*n* = 28). **(B)** UAS-*na* RNAi (*attp2* locus; *n* = 33). **(C)** UAS-*unc79* RNAi (*attp40* locus; *n* = 42). **(D–F)** *daGAL4 tubGAL80^ts^* genotypes raised at 29°C and transferred to 19°C within 2 days of eclosion. For 19°C assays, flies were subject to 12 h light: 12 h dark conditions. **(D)** *attP40* control (*n* = 46). **(E)** UAS-*na* RNAi (*n* = 27). **(F)** UAS-*unc79* RNAi (*n* = 45).

To assess the endogenous temporal requirements for the *Drosophila* NA channel complex, we used the inducible GAL4 inhibitor *tubulin*-GAL80^ts^ (*tub*GAL80^ts^) to control transgenic RNAi expression (McGuire et al., 2003; Ni et al., 2011). We combined the ubiquitous driver *daughterless*GAL4 (*da*GAL4) with *tub*GAL80^ts^ and *na* or *unc79* short-hairpin RNA interference (RNAi) constructs, maintaining flies at temperatures that are primarily permissive (19°C) or restrictive (29°C) for GAL80^ts^ function. We find that ubiquitous expression of *na* or *unc79* RNAi throughout development and adult stages at the restrictive temperature (29°C) causes severe circadian behavioral phenotypes that are comparable to *na* and *unc79* loss of function mutants (Nash et al., 2002; Lear et al., 2013). These phenotypes include decreased morning and evening anticipatory activity during light:dark (LD) conditions (**Figures 1A–C**, arrows; Supplementary Table S1, *p* < 0.05 for LD MI, *p* < 0.01 for LD EI), as well as decreased free-running rhythmicity during constant dark (DD) conditions (Supplementary Figures S1A–C and **Table 1**, 0–6% rhythmic, *p* < 0.01 compared to RNAi controls). To determine whether increased channel gene expression in adults can restore rhythmicity, we raised *tub*GAL80^ts^ RNAi crosses at 29°C and shifted adult progeny to 19°C. To minimize the effects of RNAi persistence (Bosch et al., 2016), adults were shifted to 19°C at least 2 days prior to the start of the behavioral assay. Despite this, we find that *na* and *unc79* RNAi progeny continue to exhibit multiple circadian phenotypes throughout the 19°C adult behavior assay (5 days LD; 7 days DD). Most prominently, we observe defects in DD rhythmicity (**Table 1**, < 10% rhythmic, *p* < 0.01 relative to controls) and LD evening anticipatory activity (**Figures 1D–F**, gray arrows; Supplementary Table S1, EI < 0.5, *p* < 0.01) in the 29°C development – 19°C adult shifted condition that are comparable to phenotypes observed when flies are raised and maintained at 29°C (29°C – 29°C). Notably, we find that morning behavior is not significantly disrupted in RNAi progeny in the 29–19°C condition relative to controls (**Figures 1D–F**, black arrows; Supplementary Table S1, *p* > 0.28 for MI and EI values). Thus, our data suggest that developmental expression of NA channel subunits can contribute to adult rhythmicity, but the impact on behavioral rhythms may not be uniform.

**Table 1:** Developmental expression of NA channel subunits and regulators is required for adult rhythmicity.

Genotype	Temp. (Dev. – > Adult)	Period ± SEM (Hours)			Power ± SEM			Rhythmic (%)	*n*
*daGAL4 tubGAL80^ts^*/ *attP2*	29 – > 29	22.9	±	0.0	82	±	7	97	34
*attP40/+; daGAL4 tubGAL80^ts^/+*	29 – > 29	23.1	±	0.0	63	±	8	86	28
*daGAL4 tubGAL80^ts^*/ *na RNAi (attP2)*	29 – > 29	21			2	±	1	6^∗∗^	18
*unc79 RNAi (attP40)/+; daGAL4 tubGAL80^ts^/+*	29 – > 29	NA			0.8	±	0.3	0^∗∗^	30
*daGAL4 tubGAL80^ts^*/ *Nlf-1 RNAi (attP2)*	29 – > 29	NA			1	±	1	6^∗∗^	17
*daGAL4 tubGAL80^ts^*/ *attP2*	29 – > 19	23.5	±	0.1	22	±	4	51	37
*attP40/+; daGAL4 tubGAL80^ts^/+*	29 – > 19	24.1	±	0.1	35	±	4	82	45
*daGAL4 tubGAL80^ts^*/ *na RNAi (attP2)*	29 – > 19	23.8			2	±	1	8^∗∗^	25
*unc79 RNAi (attP40)/+; daGAL4 tubGAL80^ts^/+*	29 – > 19	22.1	±	2.1	2	±	1	9^∗∗^	44
*daGAL4 tubGAL80^ts^*/ *Nlf-1 RNAi (attP2)*	29 – > 19	NA			0.7	±	0.4	0^∗∗^	25
*daGAL4 tubGAL80^ts^*/ *attP2*	19 – > 19	22.9	±	0.6	15	±	4	37	38
*attP40/+; daGAL4 tubGAL80^ts^/+*	19 – > 19	24.2	±	0.2	22	±	4	56	36
*daGAL4 tubGAL80^ts^*/ *na RNAi (attP2)*	19 – > 19	23.7	±	0.1	14	±	3	37	41
*unc79 RNAi (attP40)/+; daGAL4 tubGAL80^ts^/+*	19 – > 19	24.2	±	0.5	11	±	2	35	48
*daGAL4 tubGAL80^ts^*/ *Nlf-1 RNAi (attP2)*	19 – > 19	23.6	±	0.2	17	±	3	47	38
*daGAL4 tubGAL80^ts^*/ *attP2*	19 – > 29	23.0	±	0.0	95	±	8	97	32
*attP40/+; daGAL4 tubGAL80^ts^/+*	19 – > 29	23.5	±	0.1	89	±	9	96	23
*daGAL4 tubGAL80^ts^*/ *na RNAi (attP2)*	19 – > 29	23.6	±	0.1	75	±	6	93	30
*unc79 RNAi (attP40)/+; daGAL4 tubGAL80^ts^/+*	19 – > 29	23.4	±	0.0	89	±	8	94	31
*daGAL4 tubGAL80^ts^*/ *Nlf-1 RNAi (attP2)*	19 – > 29	23.6	±	0.1	66	±	8	85	27

*Rhythmicity analyses from adult *Drosophila* males maintained in 5–6 days LD conditions followed by 7 days DD. Temp. column indicates the temperature (°C) at which crosses were raised (Dev.) followed by the temperature of the adult behavioral assay. Power indicates that chi-squared periodogram power minus significance threshold. Significant differences from corresponding *attp* controls are indicated by asterisks (^∗∗^*p* < 0.01), as determined by Fisher’s exact test*.

**FIGURE 2:**
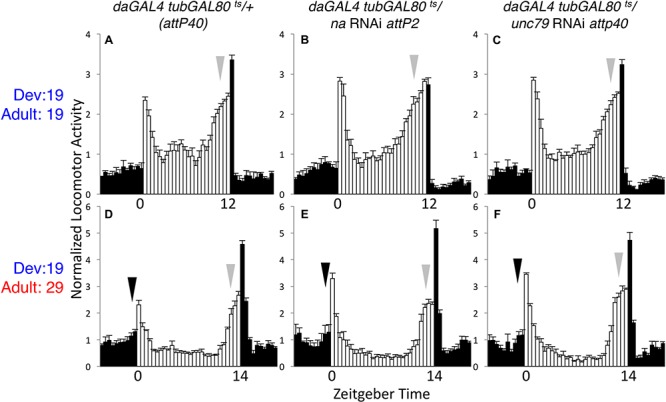
Adult-specific knockdown of *na* or *unc-79* does not strongly disrupt LD rhythmicity. Normalized activity profiles of adult males averaged over 4 days of LD conditions. Zeitgeber time indicated below each panel. White bars represent light phase activity and black bars indicate dark phase activity. Arrows indicate timing of morning (black) and evening (gray) activity, and error bars represent standard error of the mean. **(A–C)** *daGAL4 tubGAL80^ts^* genotypes raised at 19°C and maintained at 19°C throughout the behavioral assay (12 h light: 12 h dark conditions). **(A)** *attP40* RNAi insertion control (*n* = 37). **(B)** UAS-*na* RNAi (*attp2* locus; *n* = 41). **(C)** UAS-*unc79* RNAi (*attp40* locus; *n* = 48). **(D–F)** *daGAL4 tubGAL80^ts^* genotypes raised at 19°C and transferred to 29°C on the 1st day of the behavioral assay. Here, flies were subject to 14 h light: 10 h dark conditions. **(D)** *attP40* control (*n* = 27). **(E)** UAS-*na* RNAi (*n* = 38). **(F)** UAS-*unc79* RNAi (*n* = 32).

To verify that GAL80^ts^ can suppress *da*GAL4 driven RNAi phenotypes at the presumed permissive temperature, we assessed circadian behavior in flies maintained at 19°C throughout development and adult stages. Under these conditions, *unc79* and *na* RNAi flies exhibit rhythmicity comparable to controls. This includes similar LD anticipatory behavior (**Figures 2A–C**, arrows; Supplementary Table S1, *p* > 0.51 for MI and EI except where RNAi value exceeds control) and DD activity peaks (Supplementary Figures S2A–C, arrows; Supplementary Table S1, *p* > 0.18 for DD MI and EI). While DD rhythmicity is typically weaker at 19°C than at 29°C in our assays, rhythmicity measurements in the 19°C development – 19°C adult condition are similar between RNAi strains and the corresponding controls (**Table 1**, > = 35% rhythmic, *p* > 0.07 between RNAi and control strains). To determine whether adult-driven expression of NA channel subunits is required for rhythmic behavior, we raised *unc79* and *na* RNAi flies at 19°C and shifted them to 29°C as adults. Surprisingly, we observe that these RNAi flies retain robust rhythmic behaviors, including LD and DD evening activity peaks (**Figures 2D–F**, gray arrows; Supplementary Figures S2D–F, gray arrows; Supplementary Table S1, *p* > 0.89 except when RNAi > control), and free-running DD rhythmicity (**Table 1**, > 90% rhythmic, *p* > 0.60). This suggests that sustained adult expression of NA channel subunits is not required for adult behavioral rhythms. While RNAi strains raised at 19°C exhibit significant rhythmicity throughout the 29°C behavioral assay (see **Table 1**), we observe some differences in DD morning behavior relative to controls. Control strains in the 19–29°C condition retain a substantial morning activity peak on the first 2–3 days of DD (Supplementary Figure S2D, black arrows; Supplementary Table S1), while both *na* and *unc79* RNAi strains exhibit decreased morning behavior beginning on DD Day 2 (Supplementary Figures S2E,F, black arrows; Supplementary Table S1, DD Day 2 MI < = 0.31, *p* < 0.01 relative to controls).

### Developmental Expression of Nlf-1 Promotes Adult Rhythmicity

**FIGURE 3:**
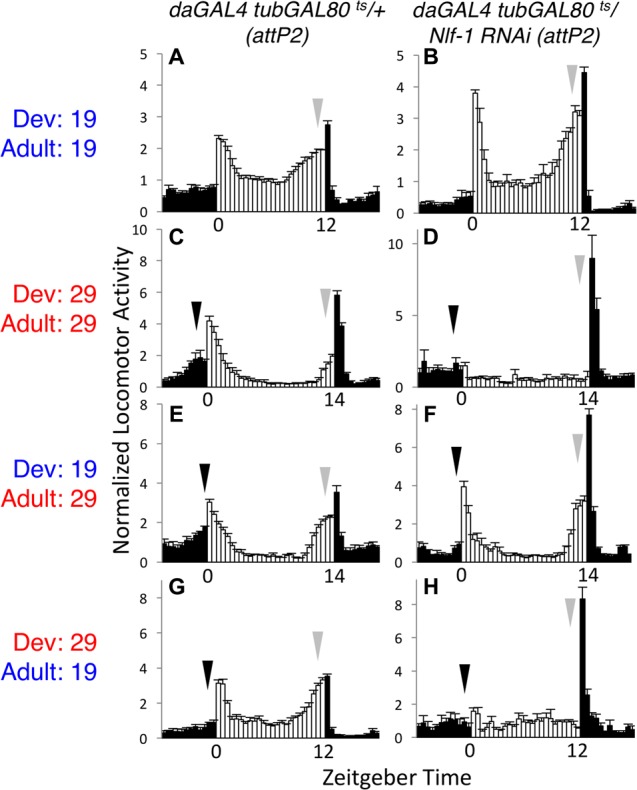
Developmental requirement for *Nlf-1* for adult rhythmic behavior. Normalized activity patterns of adult males averaged over 4 days of 12 h light: 12 h dark conditions (19°C behavioral assays) or 14 h light: 10 h dark conditions (29°C behavioral assays). Zeitgeber time indicated below each panel. White bars indicate light phase activity, black bars indicate dark phase activity, and error bars represent standard error of the mean. Where included, black arrows indicate morning activity and gray arrows evening activity. Each genotype is *da*GAL4 *tub*GAL80^ts^/+ with RNAi or control insertion as indicated. **(A,B)** Genotypes raised at 19°C and maintained at 19°C throughout the behavioral assay. **(A)** *attP2* RNAi insertion control (*n* = 39). **(B)** UAS-*Nlf-1* RNAi (*attp2* locus; *n* = 37). **(C,D)** Genotypes raised and maintained at 29°C. **(C)** *attP2* RNAi insertion control (*n* = 29). **(D)** UAS-*Nlf-1* RNAi (*attp2* locus; *n* = 27). **(E,F)** Genotypes raised at 19°C and transferred to 29°C on the 1st day of the behavioral assay. **(E)** *attP2* RNAi insertion control (*n* = 34). **(F)** UAS-*Nlf-1* RNAi (*attp2* locus; *n* = 34). **(G,H)** Genotypes raised at 29°C and transferred to 19°C within 2 days of eclosion, at least 2 days prior to the start of the behavioral assay. **(G)** *attP2* RNAi insertion control (*n* = 36). **(H)** UAS-*Nlf-1* RNAi (*attp2* locus; *n* = 28).

*Nlf-1 (aka mid1)* functions as a positive regulator of NA/NALCN/NCA channel expression and/or localization. In *Drosophila*, it has been proposed that circadian regulation of NA channel function in adult clock neurons may be mediated through rhythmic expression of *Nlf-1* (Flourakis et al., 2015). To further evaluate the requirements for *Nlf-1*, we again employed the *tub*GAL80^ts^ inducible RNAi strategy. As with *na* and *unc79*, *Nlf-1* RNAi flies raised and assayed at the permissive temperature (19°C – 19°C) exhibit rhythmic behavior comparable to controls, including LD and DD activity peaks (**Figures 3A,B**, arrows; Supplementary Figures S3A,B, arrows; Supplementary Table S1, *p* > 0.9 or RNAi MI/EI values greater than control) and DD rhythmicity (**Table 1**, *p* = 0.49). In contrast, *Nlf-1* RNAi flies raised and assessed at the restrictive temperature (29°C – 29°C) display defective rhythms (**Figures 3C,D**, arrows; Supplementary Figures S3C,D and Table S1, *p* < 0.05 except DD MI values; **Table 1**, *p* < 0.01). Moreover, like *na* and *unc79*, the adult behavioral phenotypes associated with *Nlf-1* RNAi correlate mainly with the developmental temperature. *Nlf-1* RNAi flies raised at 19°C and shifted to 29°C exhibit largely rhythmic behaviors, including intact evening activity peaks in LD and DD (**Figures 3E,F**, gray arrows; Supplementary Figures S3E,F, arrows; Supplementary Table S1), as well as robust DD rhythmicity (**Table 1**, 85% rhythmic). RNAi flies raised at 29°C and shifted to 19°C as adults exhibit significant defects in evening behavior (**Figures 3G,H**, gray arrows; Supplementary Table S1, *p* < 0.01 for LD EI) and DD rhythmicity (**Table 1**, 0% rhythmic, *p* < 0.01; Supplementary Figures S3G,H). Similar to *unc79* and *na*, *Nlf-1* RNAi strains shifted from 19 to 29°C as adults display a decrease in morning behavior relative to controls (**Figures 3E,F**, black arrows; Supplementary Figures S3E,F, black arrows; Supplementary Table S1, *p* < 0.05 for LD MI, *p* < 0.01 for DD day 2 MI). Despite this, rhythmicity over 7 days DD in *Nlf-1* RNAi flies in the 19 to 29°C condition is not significantly lower than controls (**Table 1**; *p* = 0.17).

### Developmental Expression of Endogenous Channel Regulators Promotes NA Complex Expression in Adults

**FIGURE 4:**
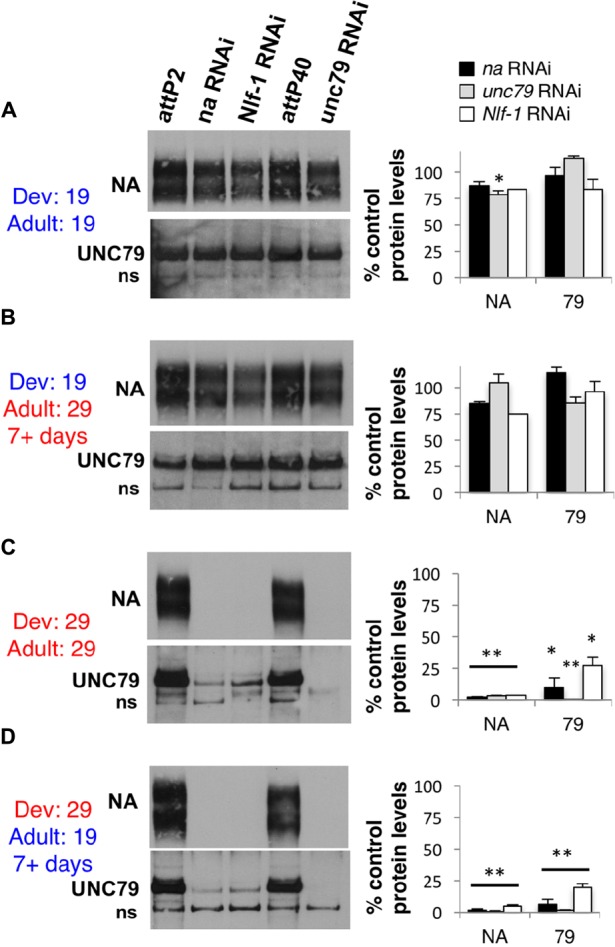
Developmental expression of *na*, *unc79*, and *Nlf-1* is sufficient to produce substantial NA and UNC79 protein expression in adults. Western blot analyses of NA and UNC79 expression performed from adult *Drosophila* head extracts. Strains were raised at 19°C or 29°C, and adult progeny were either maintained at the same temperature or shifted to the opposing temperature, as indicated (Dev = developmental temperature; Adult = adult temperature). See Section “Materials and Methods” for more details. Left panels: Representative Western blot data. All genotypes include *da*GAL4 *tub*GAL80^ts^/+ with RNAi or *attP* controls as indicated. ns = non-specific UNC79 bands (Lear et al., 2013). Right panels: Quantitation of NA and UNC79 protein levels in each RNAi genotype, as a percentage of the corresponding *attP* insertion control (*na* and *Nlf-1* RNAi constructs inserted into *attP2* locus, *unc79* RNAi into the *attP40* locus). Bars represent NA or UNC79 protein levels in strains containing *na* RNAi (black), *unc79* RNAi (gray), or *Nlf-1* RNAi (white) constructs. Error bars indicate standard error of the mean. **(A)** *daGAL4 tubGAL80^ts^*/+ genotypes raised and maintained at 19°C. **(B)** Genotypes raised at 19°C and shifted to 29°C for 7 or more days before protein extraction. **(C)** Genotypes raised and maintained at 29°C. **(D)** Genotypes raised at 29°C and shifted to 19°C for 7 or more days before protein extraction. For panels **(A,C,D)**, significant differences from *attp* controls in the same temperature condition are indicated by ^∗^*p* < 0.05 or ^∗∗^*p* < 0.01, as determined by Student’s *t*-test. For panel **(B)**, protein levels are not significantly different from the corresponding 19°C to 19°C samples (*p* > 0.13).

These behavioral data suggest a predominant developmental requirement for *na* and its key regulators. To determine how this relates to the expression of NA channel complex proteins, we performed Western blot analyses on *da*GAL4 *tub*GAL80^ts^ RNAi flies raised at either permissive (19°C) or restrictive (29°C) temperatures. We assessed protein levels for both NA and the auxiliary channel subunit UNC79, as antibodies to these two channel subunits have yielded consistent Western blot data in previous assays (Lear et al., 2013). We find that *na*, *unc79*, or *Nlf-1* RNAi flies maintained at 19°C throughout development and adult stages retain substantial expression of both NA and UNC79, with average protein levels ≥ 78% of controls (**Figure 4A**). The minor decreases in protein levels observed in some RNAi samples (**Figure 4A**, *p* < 0.01 for NA levels in *unc79* RNAi, others *p* > 0.06) may reflect residual *da*GAL4/ RNAi activity at 19°C and/or variability in the Western blot assay. In contrast, RNAi strains raised and maintained at 29°C exhibit major decreases in channel complex expression (**Figure 4C**), with minimal NA expression in all three RNAi genotypes (<4% of controls; *p* < 0.01), and UNC79 expression ranging from < 1% (*unc79* RNAi) to 27% (*Nlf-1* RNAi) of controls (*p* < 0.05). These results are consistent with previous findings in loss-of-function mutants and constitutive pan-neuronal RNAi (Lear et al., 2013; Flourakis et al., 2015). We find that RNAi strains raised at 19°C and shifted to 29°C for seven or more days during adulthood express NA and UNC79 at similar levels to the 19°C – 19°C group (**Figure 4B**; *p* ≥ 0.13). This indicates that substantial expression of NA channel complex proteins is retained in adults even when *na, unc79*, or *Nlf-1* gene expression is inhibited. For RNAi strains raised at 29°C and shifted to 19°C for a minimum of 7 days, we observe defects in protein expression comparable to the constitutive 29°C group (**Figure 4D**). Protein levels in the 29–19°C condition are significantly lower than controls (NA < 5% of controls, UNC79 < 20% of controls, *p* < 0.01) and in most cases are also significantly lower than the corresponding 19°C – 19°C samples (*p* < 0.05 except UNC79 in *Nlf-1* RNAi, *p* = 0.06). Taken together, these data suggest that much of the NA channel complex that functions in *Drosophila* adults is produced during development.

### Transgenic Expression of na in Adult Pacemaker Neurons Is Sufficient to Promote Rhythmic Behavior in the Presence of Nlf-1

**FIGURE 5:**
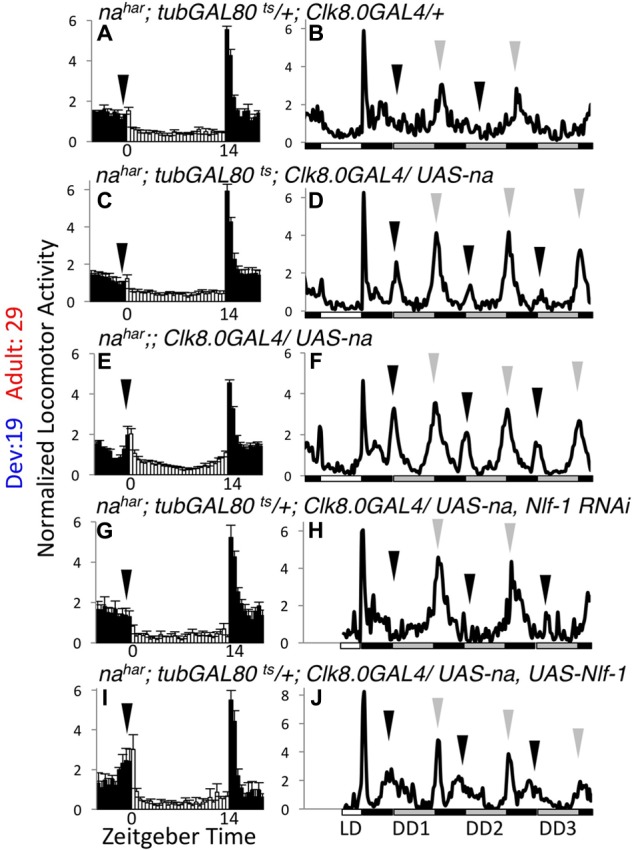
Transgenic NA rescue in adult pacemaker neurons restores DD rhythmicity. Normalized activity patterns of adult males assayed over 5–6 days of 14 h light: 10 h dark conditions (14L:10D) followed by 7 days of constant darkness (DD) at 29°C. Left panels **(A,C,E,G,I)**: Average activity patterns over 4 days of 14L:10D conditions. Zeitgeber time indicated below each panel. White bars indicate light phase activity while black bars represent dark phase activity. Error bars represent standard error of the mean, and arrows indicate timing of morning activity. In panels **(G,I)**, the 4 days used in data analysis were non-consecutive for some flies due to a ∼12 h disruption in data collection. Right panels **(B,D,F,H,J)**: Daily activity profiles over the last day of 14L:10D followed by 3 days of DD. White bars indicate light phase, while gray bars indicate subjective light phase in DD, and black bars represent dark phase or subjective dark phase. Black arrows indicate DD morning activity peak, while gray arrows indicate DD evening activity peak. In panels **(H,J)**, data is missing at the beginning of the last LD day for some flies due to the data collection disruption, so the affected time frame was excluded from those profiles. All genotypes are *na^har^;;Clk8.0*GAL4 raised at 19°C and shifted to 29°C on the 1st day of the behavior run. Strains assayed also include the following transgenes: **(A,B)** *tub*GAL80^ts^ (*n* = 54 LD; *n* = 47 DD), **(C,D)** *tub*GAL80^ts^; UAS-*na* U4 (*n* = 58 LD; *n* = 39 DD), **(E,F)** UAS-*na* U4 (*n* = 51 LD; *n* = 38 DD), **(G,H)** *tub*GAL80^ts^; UAS-*na* U4, UAS-*Nlf-1* RNAi (*n* = 26 LD; *n* = 20 DD), **(I,J)** *tub*GAL80^ts^; UAS-*na* U4, UAS-*Nlf-1-V5* (*n* = 15 LD; *n* = 12 DD).

To complement the inducible RNAi approach, we also performed tissue-specific rescue of *na^har^* mutants. For behavioral experiments, we used the broad circadian neuron driver *Clock8.0*GAL4 (*Clk8.0*GAL4) in combination with UAS-*na* and *tub*GAL80^ts^. We have previously demonstrated that constitutive *Clk8.0*GAL4 driven expression of UAS-*na* promotes robust rescue of *na* behavioral phenotypes in LD and DD conditions (Lear et al., 2005). In the inducible scheme, we find that *tub*GAL80^ts^ is quite effective at blocking *Clk8.0*GAL4 UAS-*na* rescue of *na^har^* mutant phenotypes at 19°C, as flies raised and assayed at 19°C are poorly rhythmic (**Table 2**, < 20% rhythmic, *p* = 0.11; Supplementary Figure S4, arrows; Supplementary Table S2; *p* > 0.1 except DD day 1 EI). In contrast, *na^har^; tub*GAL80^ts^/+*; Clk8.0*GAL4*/ UAS-na* flies maintained at 29°C throughout development and adulthood exhibit strong rhythmicity (**Table 2**, *p* < 0.01; Supplementary Figure S4, black arrows; Supplementary Table S2, *p* < 0.01 for LD MI and DD Day 1 MI/EI). When we restrict UAS-*na* expression to adulthood (19°C development – 29°C adult), LD behavior initially appears comparable to mutant controls (**Figures 5A,C**; black arrows; Supplementary Table S1, *p* > 0.43 for LD MI and EI). However, by the 1st day of constant darkness, locomotor activity patterns of adult rescue flies are substantially more rhythmic than mutant controls (**Figures 5B,D**, arrows; Supplementary Table S2, *p* < 0.01 for DD day 1 MI/EI), and rhythmicity over 7 days of DD is similar to constitutively expressed rescue (**Table 2**, *p* = 1.0). These data indicate that adult-driven transgenic *na* expression can promote behavioral rhythmicity. Notably, mRNA of the NA regulators *Nlf-1, unc79*, and *unc80* is endogenously expressed in the adult *Drosophila* brain (Robinson et al., 2013). To determine whether adult *Nlf-1* expression is required for adult-specific *na* rescue, we co-expressed UAS-*Nlf-1* RNAi with UAS-*na* in the 19°development – 29°adult condition. We find that RNAi knock down of *Nlf-1* in adults blocks robust adult-specific *na* behavioral rescue, with prominent decreases in DD morning behavior (**Figures 5D,H**, black arrows; Supplementary Table S2, *p* < 0.01 for DD day 1 MI) and DD rhythmicity (**Table 2**, *p* < 0.01). Moreover, co-expression of UAS-*Nlf-1* with UAS-*na* in the adult rescue scheme also alters the behavioral profile, enhancing LD morning behavior (**Figures 5C,I** and Supplementary Table S2, *p* < 0.01) while decreasing DD rhythmic power (**Table 2**, *p* < 0.01). These data suggest that adult *Nlf-1* expression is important for adult-specific production of the functional NA channel complex. Conversely, we performed development-specific rescue of *na* by raising crosses at 29°C and shifting to 19°C in adulthood. Here, developmental expression of transgenic *na* is sufficient to promote significant restoration of adult rhythmicity (Supplementary Figure S5 and **Table 2**, *p* < 0.01). However, in this condition, morning behavior is not strongly rescued (Supplementary Figures S5C,D, black arrows; Supplementary Table S2, *p* > 0.3 for MI comparisons), and DD rhythmicity is decreased relative to constitutive expression (**Table 2**; *p* < 0.01). Overall, our rescue data support a role for developmentally produced NA channel complex in the adult circadian pacemaker. These data also indicate that adult-driven channel expression may contribute in some contexts, including within the clock neurons that promote morning behavior.

**Table 2:** Transgenic rescue of NA in adult circadian neurons promotes robust DD rhythms.

Genotype	Temp. (Dev. – > Adult)	Period ± SEM (Hours)			Power ± SEM			Rhythmic (%)	*n*
*na^har^; tubGAL80^ts^; Clk8.0GAL4*	19 – > 19	24.4	±	0.6	2	±	1	6	36
*na^har^; tubGAL80^ts^; Clk8.0GAL4/ UAS-na*	19 – > 19	24.4	±	0.6	6	±	1	19^&&^	52
*na^har^;; Clk8.0GAL4/ UAS-na*	19 – > 19	24.6	±	0.1	61	±	7	89^∗∗^	28
*na^har^; tubGAL80^ts^; Clk8.0GAL4*	29 – > 29	23.5			3	±	2	8	13
*na^har^; tubGAL80^ts^; Clk8.0GAL4/ UAS-na*	29 – > 29	24.1	±	0.1	43	±	6	83^∗∗^	24
*na^har^;; Clk8.0GAL4/ UAS-na*	29 – > 29	23.8	±	0.0	42	±	5	88^∗∗^	33
*na^har^; tubGAL80^ts^; Clk8.0GAL4*	19 – > 29	24.9	±	0.5	8	±	2	27	30
*na^har^; tubGAL80^ts^; Clk8.0GAL4/ UAS-na*	19 – > 29	24.1	±	0.1	62	±	6	89^∗∗^	37
*na^har^;; Clk8.0GAL4/ UAS-na*	19 – > 29	24.0	±	0.0	64	±	7	88^∗∗^	34
*na^har^; tubGAL80^ts^; Clk8.0GAL4/ UAS-na UAS-Nlf-1 RNAi*	19 – > 29	24.8	±	0.3	21	±	6	46^&&^	13
*na^har^; tubGAL80^ts^; Clk8.0GAL4/ UAS-na UAS-Nlf1*	19 – > 29	23.9	±	0.2	26	±	8^&&^	71	7
*na^har^; tubGAL80^ts^; Clk8.0GAL4*	29 – > 19	21.5			1	±	0	2	50
*na^har^; tubGAL80^ts^; Clk8.0GAL4/ UAS-na*	29 – > 19	24.1	±	0.2	10	±	2	34^∗∗&&^	68
*na^har^;; Clk8.0GAL4/ UAS-na*	29 – > 19	24.5	±	0.2	33	±	4	66^∗∗^	62

*Rhythmicity analyses from adult *Drosophila* males maintained in 5–6 days LD conditions followed by 7 days DD. Genotypes indicated in the left column. Temp. column indicates the temperature (°C) that crosses were raised at (Dev.) followed by the temperature of the adult behavioral assay. Power indicates chi-squared periodogram power minus significance. Significant differences from the corresponding mutant controls (*na^*har*^; tubGAL80ts; Clk8.0GAL4*) and/or constitutive rescue (*na^har^; Clk8.0GAL4/UAS-na*) are indicated by ^∗^ (^∗∗^*p* < 0.01) or ^&^ (^&&^*p* < 0.01), respectively. Significance was determined using Fisher’s exact test.*.

### Developmentally Produced Transgenic NA Protein Persists in Adults with Little Degradation

**FIGURE 6:**
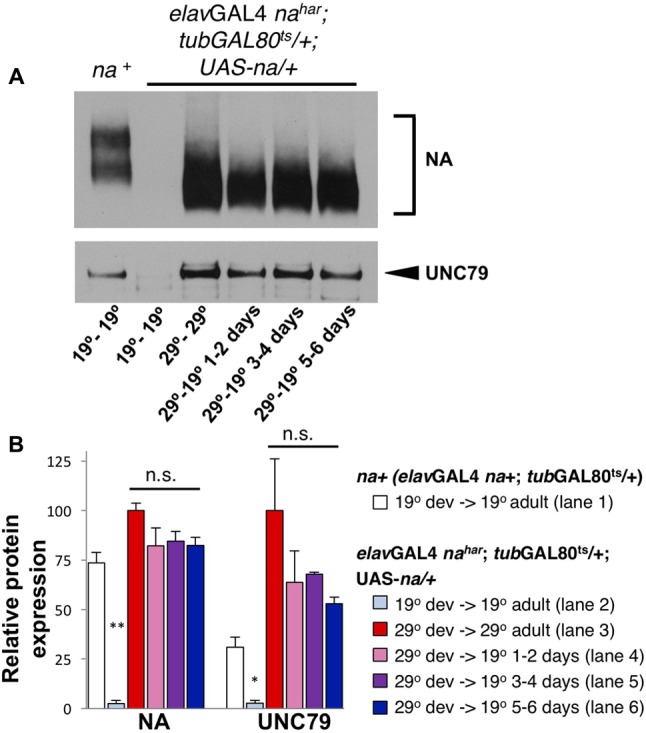
Transgenic NA protein produced during development persists into adulthood. Western blot analyses of NA and UNC79 expression from head extracts of *Drosophila* males raised at 19°C or 29°C and maintained at 19°C or 29°C as adults, as indicated. **(A)** Representative Western blot data. Lane 1 (*na+*): *elav*GAL4 *na*^+^; *tub*GAL80^ts^/ + males, raised and maintained at 19°C. Lanes 2–6: *elav*GAL4 *na^har^*; *tub*GAL80^ts^/ +; UAS-*na*/+ males, with developmental (dev) and adult temperature conditions indicated below each lane (dev temperature – adult temperature). For NA (top blot), two bands are typically observed for the endogenous protein (lane 1) while transgenic UAS-*na* produces a single band of lower molecular weight (lanes 2–6) (Lear et al., 2005). **(B)** Quantitation of NA and UNC79 levels for genotypes and conditions in panel **(A)** (see key). Bars indicate normalized protein levels, as a percentage of levels observed in *elav*GAL4 *na^har^*; *tub*GAL80^ts^/ +; UAS-*na*/+ flies in the 29°C development- 29°C adult condition (red bars; part **A**, lane 3). Error bars indicate standard error of the mean, as determined from two independent experiments. For *elav*GAL4 *na^har^*; *tub*GAL80^ts^/ +; UAS-*na*/+ flies raised and maintained at 19°C (light blue bars; panel **A**, lane 2), asterisks denote significant differences from *elav*GAL4; *tubGAL80*^ts^/ + flies maintained in the same conditions (white bars, ^∗^*p* < 0.05, ^∗∗^*p* < 0.01; panel **A**, lane 1). For *elav*GAL4 *na^har^*; *tub*GAL80^ts^/ +; UAS-*na*/+ flies raised at 29°C, adults were either maintained at 29°C (red bars; panel **A**, lane 3) or were shifted to 19°C for 1–2 days (pink bars; panel **A**, lane 4), 3–4 days (purple bars; panel **A**, lane 5), or 5–6 days (dark blue bars; panel **A**, lane 6). Protein levels in the 29°C to 19°C shifted conditions were not significantly different from the 29°C to 29°C control (n.s.; *p* > 0.08). Statistical comparisons were made using Student’s *t*-test.

If much of the NA channel complex that functions in the adult brain is produced during development, this implies that the channel complex is very stable. To address this further, we performed inducible *na* rescue experiments using the pan-neuronal driver *elav*GAL4. Notably, constitutive *elav*GAL4 driven expression of UAS-*na* restores both behavioral rhythmicity and NA channel complex expression to *na^har^* mutants (Lear et al., 2005, 2013). We find that *elav*GAL4 *na^har^*; *tub*GAL80^ts^/+; UAS*-na*/+ flies maintained at 19°C throughout development and adult stages express very little NA protein compared to a wild-type (*na+*) control (**Figure 6A**, top panel, lanes 1–2; quantified in **Figure 6B**, <4% of wild-type, *p* < 0.01). Moreover, UNC79 levels in this genotype (**Figure 6A**, bottom panel, lanes 1–2; **Figure 6B**, <10% of wild-type, *p* < 0.05) are comparable to levels previously observed in *na* loss-of-function mutants (Lear et al., 2013). Thus, GAL80ts appears highly effective at blocking *elav*GAL4 at 19°C, although we cannot exclude low level transgene expression. However, when this strain is raised and maintained at 29°C, we observe strong expression of both NA and UNC79 proteins (**Figure 6A**, lane 3). Moreover, when *elav*GAL4 *na^har^*; *tub*GAL80^ts^/+; UAS*-na*/+ flies are raised at 29°C and then shifted to 19°C as adults, we continue to observe high levels of channel complex proteins for at least 5–6 days after the temperature shift (**Figure 6A**, lanes 4–6; **Figure 6B**, 53–85% of 29°C – 29°C protein levels). Thus, the NA channel complex produced during developmental stages is indeed quite stable.

**FIGURE 7:**
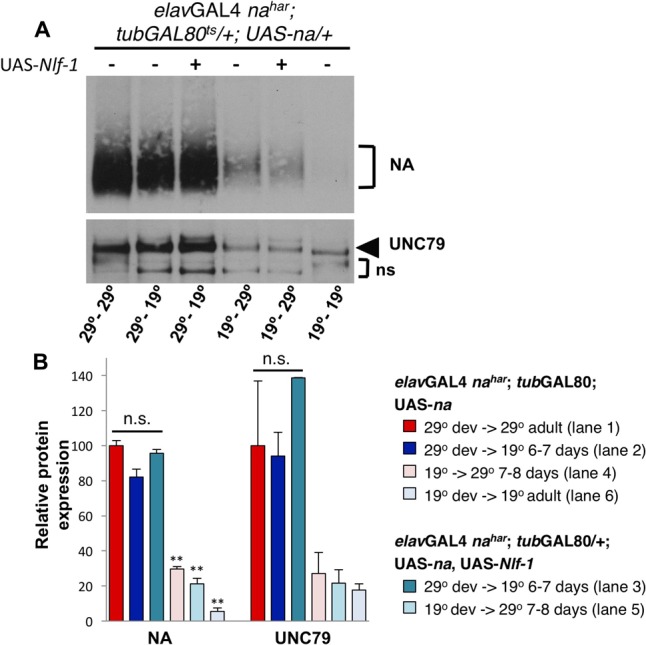
Developmental expression of UAS-*na* yields higher levels of channel complex proteins than adult-restricted expression. Western blot analyses of NA and UNC79 expression from head extracts in *Drosophila* males raised at 19°C or 29°C and maintained at 19°C or 29°C as adults, as indicated. **(A)** Representative Western blot data. All flies are *elav*GAL4 *na^har^*; *tub*GAL80^ts^/ +; UAS-*na*/+, and some strains include co-expression of UAS-*Nlf-1* (lanes 3,5) (Flourakis et al., 2015). Developmental (dev) and adult temperature conditions are indicated below each lane (dev temperature – adult temperature). Adult flies shifted from 19 to 29°C (lanes 4–5) were maintained at 29°C for 7–8 days prior to protein extraction, while flies shifted from 29 to 19°C (lanes 2–3) were maintained at 19°C for 6–7 days before extraction. **(B)** Quantitation of NA and UNC79 levels for genotypes and conditions shown in panel (**A**; see key). Bars indicate normalized protein levels, as a percentage of levels observed in *elav*GAL4 *na^har^*; *tub*GAL80^ts^/ +; UAS-*na*/+ flies in the 29°C development- 29°C adult condition (red bars; part **A**, lane 1). Error bars indicate standard error of the mean, as determined from two independent experiments. For *elav*GAL4 *na^har^*; *tub*GAL80^ts^/ +; UAS-*na*/+ shifted from 29 to 19°C, with or without UAS-*Nlf-1* (dark blue and dark teal bars; part **A**, lanes 2–3), protein levels are not significantly different from the 29°C to 29°C control (*p* > 0.08). For *elav*GAL4 *na^har^*; *tub*GAL80^ts^/ +; UAS-*na*/+ shifted from 19 to 29°C in adulthood, with or without UAS-*Nlf-1* (dark blue and dark teal bars; part **A**, lanes 2–3), NA protein levels are significantly different from the 29°C to 29°C control (^∗∗^*p* < 0.01). UNC79 protein levels in the 19°C to 29°C shifted conditions are < 30% of 29°C to 29°C control, but this difference does not reach significance. Statistical comparisons were made using Student’s *t*-test.

To compare the capacity to produce the NA channel complex during development vs. adulthood, we restricted transgenic UAS-*na* rescue to either condition and compared protein expression levels. Similar to above, we observe that developmentally restricted UAS-*na* expression yields high levels of NA and UNC79 protein in adults assayed 6–7 days later (**Figure 7A**, lanes 1–2; **Figure 7B**, red vs. dark blue bars). Co-expression of *Nlf-1* during development does not significantly alter NA or UNC79 levels in adults (**Figure 7A**, lanes 2–3; **Figure 7B**, dark blue vs. dark teal bars, *p* > 0.16). In contrast to developmental expression results, transgenic rescue of *na* in adults (*elav*GAL4 *na^har^*; *tub*GAL80^ts^/+; UAS*-na*/+ 19°C shifted to 29°C for 7–8 days) produces only modest levels of NA and UNC79 protein (**Figure 7A**, lane 4; **Figure 7B**, pink bars, ∼27–30% of 29°C – 29°C levels, *p* < 0.01 for NA). Thus, transgenic expression of UAS-*na* in the adult nervous system can promote production of the channel complex, but likely at much lower levels than developmentally restricted or constitutive expression of the transgene. Based on our behavioral results (**Figures 5I,J**), we also considered whether *Nlf-1* could be a limiting factor for the production of new channel complex in adults. However, we find that co-expression of *Nlf-1* with *na* in adult neurons does not produce a discernable increase in NA or UNC79 levels in head extracts (**Figure 7A**, lanes 4–5; **Figure 7B**, pink vs. light teal bars). This suggests that other factors independent of *Nlf-1* expression levels limit the production of the NA channel complex in the *Drosophila* adult nervous system.

We also pooled data from developmentally restricted transgenic rescue experiments in order to estimate the half-life of the NA channel complex. We compared protein expression within *elav*GAL4 *na^har^*; *tub*GAL80^ts^/+; UAS*-na*/+ flies raised and maintained at 29°C (i.e., 29°C – 29°C) to those shifted from 29°C development to 19°C for 5–7 days of adulthood (e.g., **Figure 6A**, lane 6; **Figure 7A**, lane 2). From these pooled data, we find a small but significant decrease in NA expression levels in 29°C to 19°C head extracts as compared to the constitutive 29°C to 29°C samples (Supplementary Table S3, 86.8% of 29–29°C levels; *p* < 0.05). For UNC79, we observe greater variability in expression, with a larger but non-significant decrease in expression in the 29–19°C extracts (Supplementary Table S3, 73.7%; *p* = 0.2). We used the more consistent difference in NA expression levels to estimate protein half-life. Assuming an average time difference for the 29–19°C condition of 6 days (range 5–7 days), our calculation yields a half-life of ∼29 days for NA protein (see Materials and Methods). This half-life estimation for NA combined with the non-significant change in UNC79 levels confirms that the NA ion channel complex is likely very stable in the *Drosophila* nervous system.

## Discussion

Several previous studies have demonstrated the importance of the *Drosophila* NA ion channel in promoting adult circadian pacemaker neuron function and adult behavioral rhythms (Lear et al., 2005, 2013; Flourakis et al., 2015). Thus, we were initially surprised to find that developmental RNAi knockdown of channel subunits or the *Nlf-1* regulator causes substantial defects in adult rhythmic behaviors comparable to strong loss-of-function mutants. In contrast, limiting RNAi knockdown of channel components or *Nlf-1* primarily to adulthood is associated with only minor behavioral phenotypes (**Figures 1**–**3** and **Table 1**). Our analyses of channel complex expression indicate that much of the endogenous NA channel complex present in adults is likely produced during development (**Figure 4**). Similarly, developmentally restricted *na* rescue promotes the production of channel complex proteins that persist well into adulthood (**Figures 6**, **7**). From these assays, we estimate that NA protein half-life may exceed 20 days (Supplementary Table S3). Large-scale proteomic studies in mammals indicate that only a small fraction of proteins exhibit half-lives in this range (Schwanhausser et al., 2011; Boisvert et al., 2012; Cohen et al., 2013). The known subunits of the NA/NALCN channel complex are each relatively large proteins (∼200–370 KDa), predicting a minimum complex size near 900 KDa. While research from mammals indicates that turnover rate does not correlate with protein size (Boisvert et al., 2012), a recent report suggests that long-lived proteins are more prevalent within large multi-protein complexes (Toyama et al., 2013). Additional studies will be required to more accurately quantify the half-life of the NA channel complex in *Drosophila* neurons, and to determine whether NALCN complexes in other species exhibit similar stability. Notably, several proteins that function in the circadian system display similar stability between *Drosophila* and mammals, including PERIOD proteins and the BK ion channel (Allada and Chung, 2010; Colwell, 2011; Pegoraro and Tauber, 2011).

We find that transgenic RNAi targeting of *Drosophila na*, *unc79*, or *Nlf-1* using the pan-cellular driver *da*GAL4 is highly effective at decreasing expression of the NA channel complex, consistent with previous reports (Lear et al., 2013; Flourakis et al., 2015). We have also combined transgenic RNAi with the inducible GAL80^ts^ system (McGuire et al., 2003), similar to approaches used in several previous *Drosophila* studies (Duvall and Taghert, 2012; Zhang et al., 2013; Petsakou et al., 2015). Our behavior and expression data indicate that GAL80^ts^ is very effective at suppressing *da*GAL4 activity at 19°C but ineffective at 29°C, although minor decreases in NA expression levels in the 19°C – 19°C condition (**Figure 4A**) could reflect some leakiness of the system. For our developmental RNAi experiments (29°C development- 19°C adult), we shifted progeny to the permissive temperature (19°C) for at least 2 days prior to the behavioral assay to minimize concerns of RNAi persistence (Bosch et al., 2016). We also complemented RNAi experiments through the use of temporally restricted *na* rescue, again using the GAL80^ts^ system. Again, behavioral assays (*Clk8.0*GAL4) and expression data (*elav*GAL4) suggest strong suppression of GAL4 by GAL80^t^*^s^* at 19°C but not at 29°C. While we cannot exclude some leaky transgene expression at 19°C or partial suppression at 29°C, these effects (if any) appear minimal (see **Figure 6** and **Table 2**) and thus are unlikely to account for our rescue findings. The parallel use of inducible RNAi and rescue approaches generally produced consistent results, with some exceptions. We observe that flies subject to adult-specific RNAi exhibit much stronger DD behavioral rhythmicity (**Table 1**) than *na* mutants in which NA expression is restricted to development (**Table 2**). Several differences in the experimental approaches used could account for this discrepancy. Behavioral assays for adult-specific knockdown were performed at 29°C while adults subject to developmental-rescue were assayed at 19°C, and we consistently observe stronger DD rhythmicity in control strains at 29°C than at 19°C (**Table 1**). Moreover, the expression pattern of the *Clk8.0*GAL4 driver used for rescue experiments has not been fully evaluated during larval and pupal development, thus it may not be strongly expressed in all relevant clock neurons during key developmental stages (Lear et al., 2005). We also observe a discrepancy between developmental RNAi and adult-specific rescue results. Developmental RNAi data suggest that adult expression of *na* or its regulators is not sufficient for rhythmicity (**Table 1**), yet we find that transgenic UAS-*na* rescue in adult clock neurons can promote strong behavioral rhythms (**Table 2**). Here, we hypothesize that endogenous *na* expression is normally a limiting factor for the production of the channel complex in adults. Thus, overexpression of UAS-*na* in adults may increase levels of the functional channel complex more effectively than simply restoring endogenous gene expression (compare **Figures 4D**, **7**). Importantly, our ability to rescue *na* mutant phenotypes in adults indicates that developmental loss of *na* does not disrupt neuronal structure or function in a permanent manner. As NA/NALCN channels are known to be important for promoting excitability, we hypothesize that adult transgenic expression of NA can correct excitability defects in mature *na* mutant neurons. Recently, both dominant and recessive mutations in human NALCN have been associated with severe neurological conditions, which are typically diagnosed after birth (Cochet-Bissuel et al., 2014; Aoyagi et al., 2015; Chong et al., 2015; Gal et al., 2016). Thus, the potential to alter the function of mature neurons in patients with *NALCN* mutations could have substantial clinical relevance (Snowball and Schorge, 2015).

Our data also provide additional insight into the role of *Nlf-1* in NA regulation, suggesting a transient requirement for *Nlf-1* during the production of the NA channel complex. We find that *Nlf-1* expression is normally required developmentally, when much of the endogenous channel complex is likely being produced. However, when we transgenically rescue NA expression in *na* mutant adults, we then observe a requirement for adult-driven *Nlf-1* (**Figure 5** and **Table 2**). Thus, the requirement for *Nlf-1* expression correlates with the production of new channel complex. This is consistent with a model in which *Drosophila* NLF-1 functions within the endoplasmic reticulum to promote proper formation and/or localization of the channel complex (Xie et al., 2013; Flourakis et al., 2015), although additional studies will be required to confirm this. Our findings also suggest that the NLF-1 protein may not be as stable as the channel complex itself, since developmental expression of *Nlf-1* is not sufficient to promote robust adult-specific UAS-*na* rescue (**Figure 5** and **Table 2**). However, direct assessments of NLF-1 protein expression will be needed to definitively assess the stability of this protein.

Loss of *na, unc79*, or *Nlf-1* function is associated with disruptions in morning and evening activity peaks and free-running rhythmicity (Lear et al., 2005, 2013; Ghezzi et al., 2014; Flourakis et al., 2015). Here, we find that developmental loss of *na* or its regulators strongly disrupts evening behavior and DD rhythmicity, while adult-specific loss of channel expression causes at least some defects in morning behavior. Previous data have indicated that NA functions within the DN1p circadian neuron subgroup to promote morning behavior (Zhang L. et al., 2010), and these clock cells have been shown to exhibit rhythmic expression of *Nlf-1* and rhythmic channel activity (Flourakis et al., 2015). Thus, it is possible that the NA channel complex is less stable in the DN1p subset of clock neurons than it is in the rest of the circadian pacemaker or in most other neurons in the brain. Western blot assays are not sensitive enough to detect NA expression differences in specific groups of circadian pacemaker neurons (data not shown), and attempts to visualize channel complex expression in individual *Drosophila* neurons have proven difficult (Lear et al., 2005). Notably, *Nlf-1* mRNA expression is highly enriched in the adult DN1p neurons (Flourakis et al., 2015), yet we find that overexpression of this gene in adult clock neurons can still enhance morning behavior (**Figure 5I**). Thus, NLF-1 could play a unique, sustained role in promoting NA channel complex production in adult DN1p neurons.

The molecular circadian clock regulates cellular output components through rhythmic transcription as well as post-transcriptional mechanisms (Lim and Allada, 2013; Partch et al., 2014). Several ion channel genes have been shown to exhibit daily expression rhythms at the transcript and/or protein level (Colwell, 2011). For some components of circadian neuronal output, it may be difficult or inefficient to produce sufficient amounts of new functional protein on a daily basis. We find that NA channel complex proteins exhibit much greater stability than we would expect if daily synthesis and recycling fully account for circadian regulation of the channel. Nonetheless, if the channel complex is less stable in the DN1p subset of clock neurons than other neurons in the *Drosophila* brain, then rhythmic production of the channel complex may contribute to rhythmic output in these cells. Based on our findings, we propose that additional post-translational mechanisms are involved in clock regulation of NA activity in the DN1p as well as other *Drosophila* pacemaker neurons. As different subsets of clock neurons may exhibit distinct patterns of neuronal activity (Liang et al., 2016), the circadian regulatory mechanisms influencing NA channel function may also vary among pacemaker neurons. Moreover, while NA is thought to function primarily downstream of the circadian clock, loss of *na* function does alter molecular clock oscillations after several days in DD (Lear et al., 2005). This is consistent with other findings that circadian neuron activity impacts molecular clock function (Nitabach et al., 2002; Mezan et al., 2016; Sabado et al., 2017). Based on the stability of the NA channel complex, we predict that developmental expression of *na* would be sufficient for normal clock oscillations in adult pacemaker neurons. However, it would be interesting to determine whether clock disruptions occur in some pacemaker cell groups owing to differential requirements for adult *na* expression.

## Author Contributions

BL, BA, and DM initiated the project and designed experiments. DM and BL generated *Drosophila* reagents and performed behavioral assays. DM, SH, and BL performed Western blot experiments. BL, SH, DM, and BA wrote and edited the manuscript.

## Conflict of Interest Statement

The authors declare that the research was conducted in the absence of any commercial or financial relationships that could be construed as a potential conflict of interest.
