# Supplementary material for: The Narrow Abdomen Ion Channel Complex Is Highly Stable and Persists from Development into Adult Stages to Promote Behavioral Rhythmicity
Source: Front Cell Neurosci. 2017 Jun 6;11:159. doi: 10.3389/fncel.2017.00159 (PMC5459923; doi:10.3389/fncel.2017.00159)
Supplement: Supplementary file 4 [file Data_Sheet_1.pdf]

Supplementary Material

The Narrow Abdomen ion channel complex is highly stable and persists from development into adult stages to promote behavioral rhythmicity

Devon L. Moose[1], Stephanie J. Haase[2], Benjamin T. Aldrich[1], Bridget C. Lear[1,2*]

*** Correspondence:** Bridget C. Lear
bridget-lear@uiowa.edu

1.1 Supplementary Figures

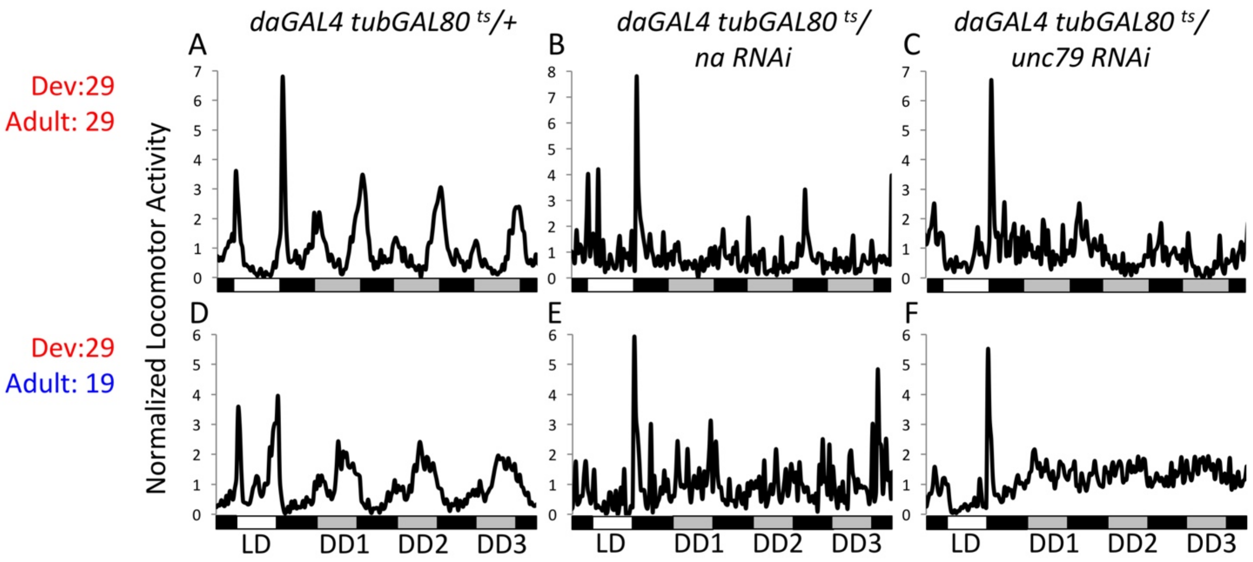

Supplemental Figure 1. Developmental knockdown of *na* and *unc-79* impairs adult DD rhythmicity. Normalized average activity profiles of male *Drosophila* over one day light:dark conditions (LD) followed by three days constant darkness (DD 1-3, as indicated). White bars indicate light phase, gray bars indicates subjective light phase during DD, and black bars indicate dark phase or subjective dark phase. All strains are *da*GAL4 *tub*GAL80^{ts} combined with the RNAi or control insertions as indicated. (A-C) Genotypes raised at 29°C and maintained at 29°C throughout the behavioral assay. For 29°C LD assays, flies were subject to 14 hour light: 10 hour dark conditions. (A) *attP40* RNAi insertion control (n=27). (B) UAS-*na* RNAi (*attp2* locus; n=23). (C) UAS-*unc79* RNAi (*attp40* locus; n=34). (D-F) Genotypes raised at 29°C and transferred to 19°C within two days of eclosion. For 19°C behavioral assays, flies were subject to 12 hour light: 12 hour dark conditions followed by constant darkness. (D) *attP40* control (n=45). (E) UAS-*na* RNAi (n=26). (F) UAS-*unc79* RNAi (n=44).

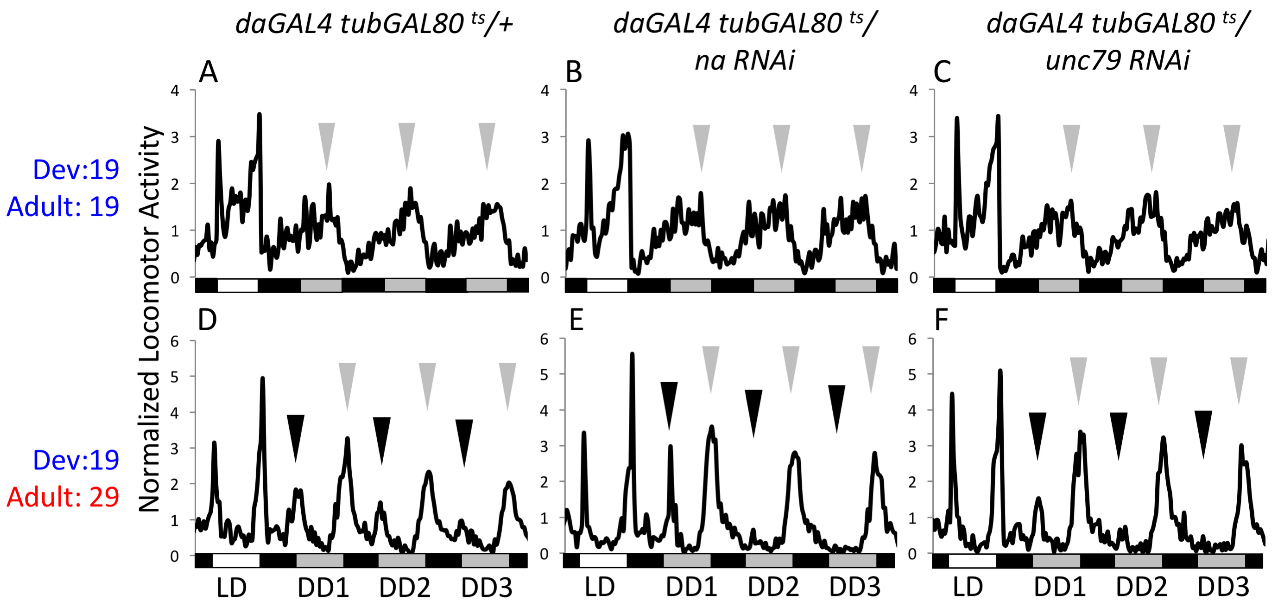

Supplemental Figure 2. Adult knockdown of *na* or *unc-79* reduces morning activity peak in DD. Normalized average activity patterns of adult males over the last day of LD followed by DD days 1-3, as indicated. Black bars indicate dark phase or subjective dark phase, white bar indicates light phase, and gray bars indicate subjective light phase. (A-C) *da*GAL4 *tub*GAL80^{ts} strains raised and maintained at 19°C. Gray arrows indicate evening activity peak. (A) *attP40* control (n=36). (B) *UAS-na* RNAi (n=41). (C) UAS-*unc79* RNAi (n=48). (D-F) Crosses raised at 19°C and progeny transferred to 29°C on the first day of the behavioral assay. Black arrows indicate normal timing of DD morning behavior. (D) *attP40* control (n=26). (E) *UAS-na* RNAi (n=36). (F) UAS-*unc79* RNAi (n=32).

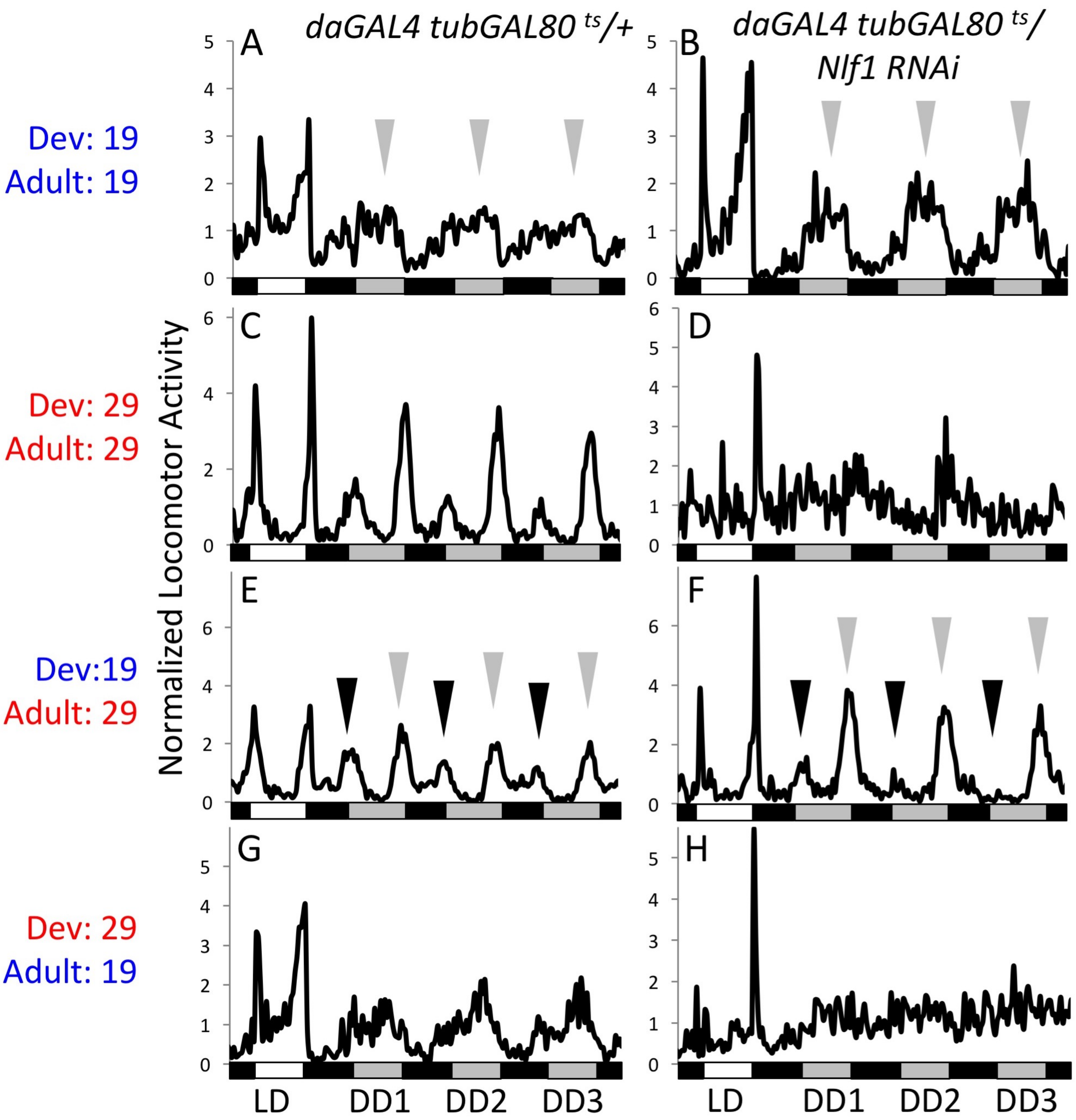

Supplemental Figure 3. Behavioral effects of developmental vs. adult specific knockdown of *Nlf-1*. Normalized average activity profiles of adult males over the last day of LD followed by three days DD, as indicated above. Black bars indicate dark phase, white bar indicates light phase, and gray bars indicate subjective light phase. Where included, black arrows indicate DD morning activity and gray arrows indicate DD evening activity. Left panels (A,C,E,G): *da*GAL4 *tub*GAL80^{ts}/ *attP2* control. Right panels (B,D,F,H): *da*GAL4 *tub*GAL80^{ts}/ *Nlf-1* RNAi. (A-B) Strains raised and maintained at 19°C (n >= 37 LD). (C-D) Strains raised and maintained at 29°C (n >= 22). (E-F) Strains raised at 19°C and transferred to 29°C on the first day of the behavioral assay (n=34). (G-H) Strains raised at 29°C and transferred to 19°C within two days of eclosion (n>= 28).

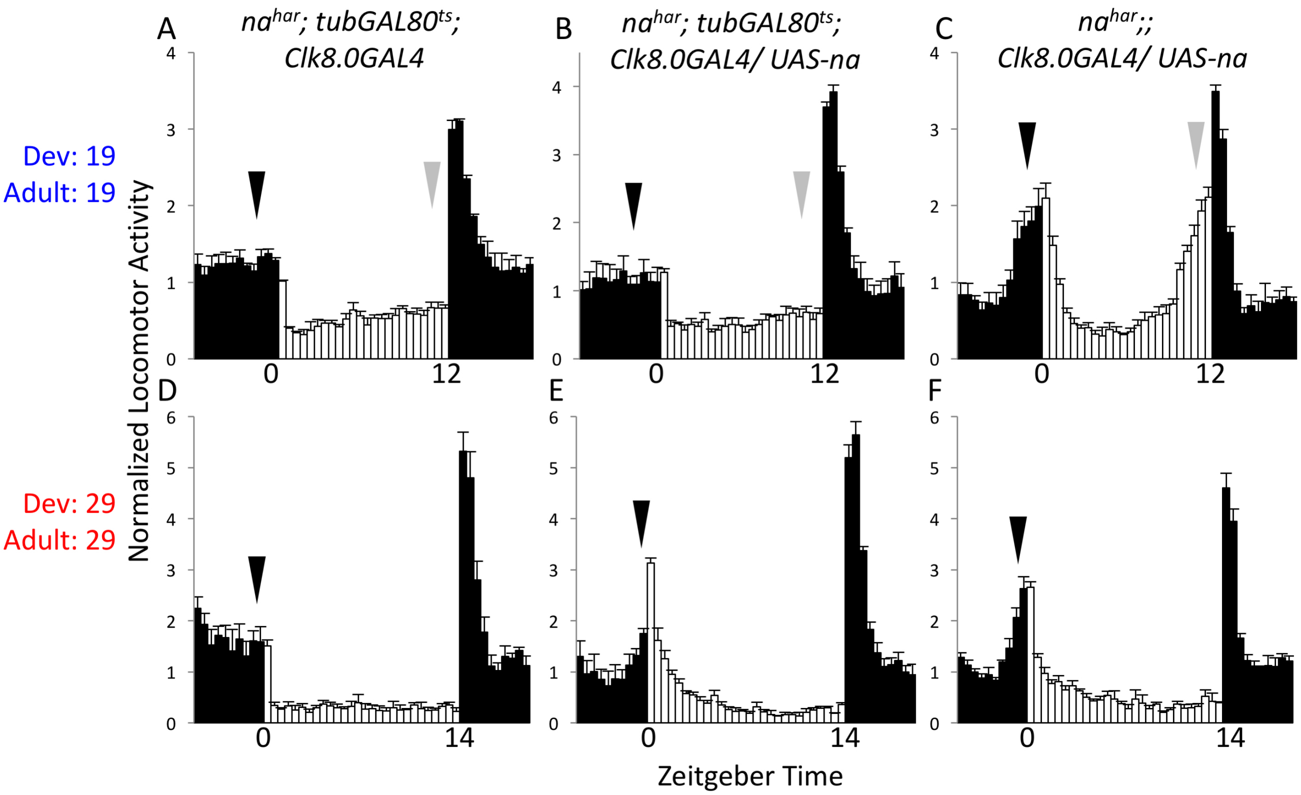

Supplemental Figure 4. GAL80^{ts} suppresses circadian rescue of *na* mutant behavior at 19° C but not 29° C. Normalized activity patterns of adult males averaged over four days of light:dark (LD) entrainment conditions. Zeitgeber time indicated below each profile. White bars indicate light phase activity, black bars represent dark phase activity, and error bars represent standard error of the mean. Black arrows indicate timing of morning anticipatory behavior, while gray arrows represent evening anticipatory behavior. All genotypes are *na^{har}; Clk8.0GAL4/+* with additional transgenes indicated as follows. (A-C) Strains raised and maintained at 19°C. For 19°C behavioral assays, flies were subject to 12 hour light: 12 hour dark conditions. (A) *tub*GAL80^{ts} (n= 36). (B) *tub*GAL80^{ts}; UAS-*na* U4 (n= 47). (C) UAS-*na* U4 (n= 29). (D-F) Strains raised and maintained at 29°C. For 29°C behavioral assays, flies were subject to 14 hour light: 10 hour dark conditions. (D) *tub*GAL80^{ts} (n= 41). (E) *tub*GAL80^{ts}; UAS-*na* U4 (n= 54). (F) UAS-*na* U4 (n= 50).

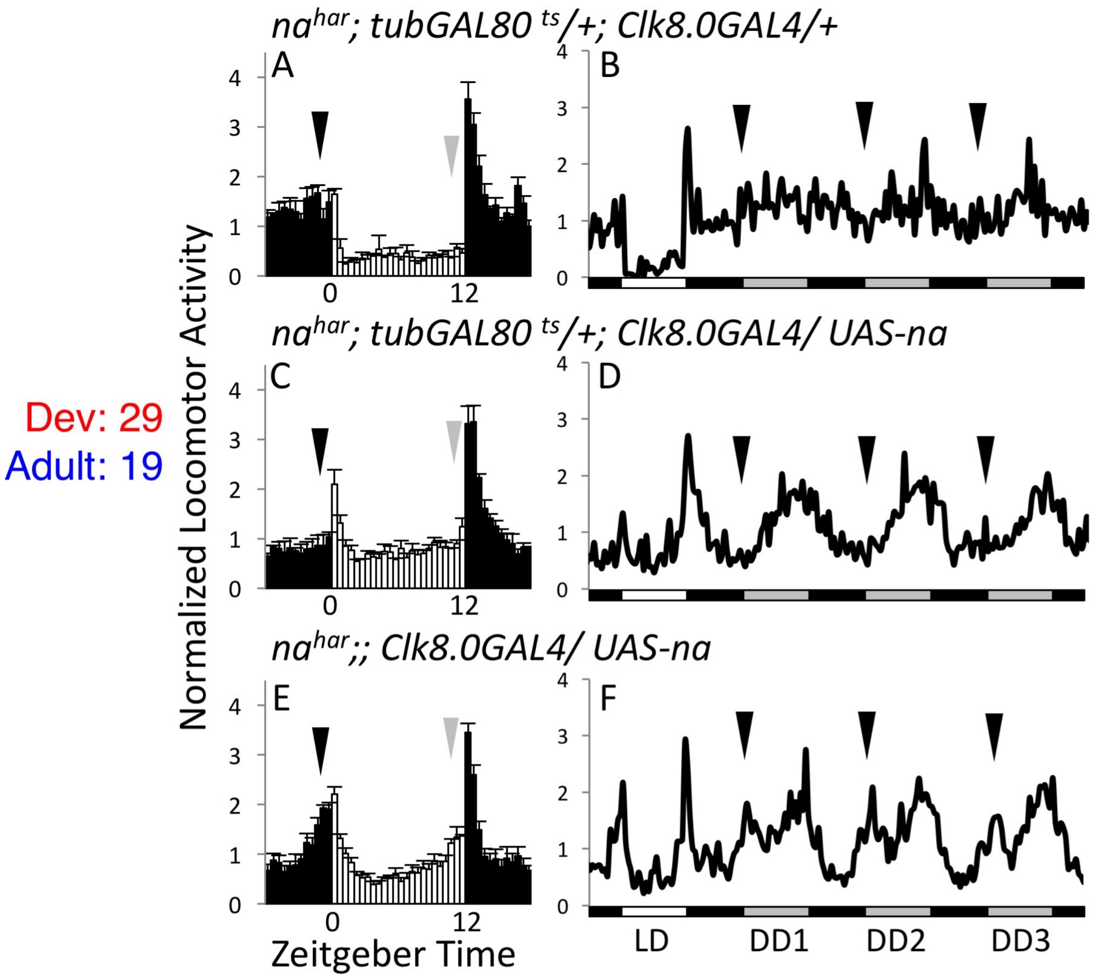

Supplemental Figure 5. Developmental expression of transgenic NA promotes adult rhythmicity. Normalized activity patterns of adult males assayed over five days of 12 hour light: 12 hour dark conditions (LD) followed by constant darkness (DD) at 19°C. Where included, black arrows indicated morning activity peak while gray arrows indicate evening activity. Left panels (A,C,E): Activity patterns averaged over the last four days of LD. Zeitgeber time indicated below each profile. White bars indicate light phase activity while black bars represent dark phase activity. Error bars represent standard error of the mean. Right panels: Daily activity profiles over the last day of LD followed by three days of DD (DD1-3). White bars indicate light phase, gray bars indicate subjective light phase in DD, and black bars represent dark phase/ subjective dark phase. All genotypes are na^{har}*; Clk8.0*GAL4/+ raised at 29°C and shifted to 19°C within two days of eclosion (29°C -> 19°C). Strains assayed also include the following transgenes: (A-B) *tub*GAL80^{ts} (n= 61 LD; n=57 DD), (C-D) *tub*GAL80^{ts}; UAS-*na* U4 (n= 80 LD; n= 74 DD), (E-F) UAS-*na* U4 (n= 61 LD; n= 66 DD).
